# Survival in children requiring chronic renal replacement therapy

**DOI:** 10.1007/s00467-017-3681-9

**Published:** 2017-05-15

**Authors:** Nicholas C. Chesnaye, Karlijn J. van Stralen, Marjolein Bonthuis, Jérôme Harambat, Jaap W. Groothoff, Kitty J. Jager

**Affiliations:** 1 ESPN/ERA-EDTA Registry, Department of Medical Informatics, Academic Medical Center, Amsterdam Public Health Research Institute, University of Amsterdam, Amsterdam, The Netherlands; 2 Spaarne Gasthuis Academie, Spaarne Gasthuis, Hoofddorp, Netherlands; 3 Department of Pediatrics, Bordeaux University Hospital and INSERM U1219, Bordeaux, France; 4 Department of Pediatric Nephrology, Emma Children’s Hospital AMC, Amsterdam, Netherlands

**Keywords:** End-stage renal disease, Mortality risk, Registry data, Global disparity, Outcomes, Children

## Abstract

Survival in the pediatric end-stage renal disease (ESRD) population has improved substantially over recent decades. Nonetheless, mortality remains at least 30 times higher than that of healthy peers. Patient survival is multifactorial and dependent on various patient and treatment characteristics and degree of economic welfare of the country in which a patient is treated. In this educational review, we aim to delineate current evidence regarding mortality risk in the pediatric ESRD population and provide pediatric nephrologists with up-to-date information required to counsel affected families.

## Introduction

Approximately nine in every 1 million children <20 years of age in the developed world require renal replacement therapy (RRT) treating end-stage renal disease (ESRD) [1]. Mortality risk in these children is multifactorial owing to the complex nature and multiple causes of ESRD in this population and is at least 30 times higher than that of healthy peers [2, 3]. Although other patient-related outcomes, such as growth, psychosocial development, and quality of life (QoL) are of major importance, prolonging patient survival may be arguably the most relevant clinical goal. As ESRD in children is a rare condition, the statistical power needed to accurately assess (risk factors related to) survival has been limited. Over the past years, various (inter)national registries have provided sufficient data to advance epidemiological research and expand the evidence regarding outcomes and treatment guidelines for this population. In this review, we delineate the current evidence base regarding mortality risk in the pediatric RRT population and provide pediatric nephrologists with up-to-date data to counsel affected families.

## Improvements in patient survival

Since the introduction of the first pediatric chronic RRT programs during the 1960s, substantial advances in renal medicine have been achieved (Table 1) [17, 18], and survival has improved significantly, especially in the youngest patients. Historic registry data from Australia and New Zealand (ANZDATA registry) cite a 10-year mortality rate of 110 deaths per 1000 patient-years during the 1960s, which was halved with each subsequent decade, stabilizing at 18 deaths per 1000 patient-years during the 1990s [19]. In European dialysis patients, the 5-year mortality risk decreased by 36% between 1980–1984 and 1995–2000 and by 79% in the subgroup of patients aged 0–4 years [20]. In the USA, dialysis survival improved from 1990 to 2010, with each 5-year increment decreasing mortality by 12% in children >5 years and by 20% in children <5 years [21]. Most neonates and infants placed on dialysis now survive long enough to reach the minimum age and body weight required for successful transplantation [22, 23].

**Table:** **Table 1** Key developments in renal medicine

Key developments	Year
Hemodiafiltration [5] Portable PD devices [7]	1967 1970s
Home HD programs [6] Continuous ambulatory peritoneal dialysis [4] On-line proportioning of bicarbonate buffer for dialysis [10] Addition of amino acids to dialysate [11]	1971 1975 1977 1980s
Y-set catheter connection for PD [9] Recombinant human erythropoietin [12]	1983 1985
Recombinant growth hormone therapy in children [13]	1994
Improved predialysis care [8] Increased percentage of pre-emptive Tx [14]	Ongoing Ongoing
Immunosuppressive drugs [15]	Ongoing
Nutrition [16]	Ongoing

*HD* hemodialysis,* PD* peritoneal dialysis,* Tx* transplantation.

Posttransplant survival has also improved over time. The mortality risk in first renal transplant recipients in Europe decreased by 42% from 1995 to 2000 compared with 1980 to 1984 [20]. Between 1990 and 2010 in the USA, each additional calendar year led to a 3% decrease in mortality risk, which was 5% for children <5 years of age. Improvements were most pronounced during the first year posttransplant [24]. The 5-year survival for deceased donor recipients improved from 91.2% during 1987–1995 to 96.4% during 2005–2013 and from 95.1% to 97.1% for living-donor recipients [25]. The overall 5-year survival for pediatric RRT patients at present is ∼90% [3, 26–30] and is similar across high-income countries (Table 2). In Europe, survival ranges from 82 to 96% at 10 years and from 76 to 89% at 20 years. Long-term survival probabilities for European patients are presented by age group and initial treatment modality in Table 3 (personal communication; ERA-EDTA Registry, 25 January 2017).

**Table 2:** Five-year crude survival probabilities of pediatric renal replacement therapy (RRT) patients by country and period

Country/area	Period	Survival
Australia and New Zealand	1963–2002	83%
United States	2004–2008	89%
Canada	1992–2007	92%
Europe^1^	2009–2011	94%
Japan	2006–2011	92%
Taiwan^2^	1995–2004	88%

^1^Four-year survival probability

^2^ Incident dialysis patients only

**Table 3:** Long-term crude survival for patients beginning renal replacement therapy (RRT) between 1990 and 2014 by age group and initial treatment modality, using European Renal Association–European Dialysis and Transplant Association (ERA-EDTA) data for Austria, Bosnia and Herzegovina, Denmark, Spain, Finland, France, Greece, Iceland, The Netherlands, Norway, Romania, Serbia, Sweden, and Scotland (personal communication; Anneke Kramer, 25 January 2017)

	5-yr	10-yr	15-yr	20-yr
Overall	94%	90%	87%	83%
Age				
0–1	85%	82%	79%	76%
2–5	92%	88%	83%	81%
6–12	95%	93%	90%	85%
13–18	95%	92%	88%	85%
First RRT modality				
HD	94%	90%	86%	82%
PD	92%	88%	85%	82%
Tx	97%	96%	93%	89%

*HD* hemodialysis,* PD* peritoneal dialysis,* Tx* transplantation

## Factors associated with mortality

### Age

Age at dialysis initiation is a key determinant of patient survival. Registry data consistently shows that compared with adolescents, mortality risk is approximately four times higher in children <5 years of age at dialysis initiation, and 1.5 times higher in children >5 years of age [3, 19, 21, 31]. Mortality risk remains the highest in neonatal and infant dialysis patients [31, 32], who are technically challenging to treat due to small body size, a high risk of infection, difficulties in nutrition and growth, and a high prevalence of (severe) comorbidities [33, 34]. These challenges and a perceived unacceptable quality of life are important factors in the decision to withhold or withdraw treatment in some of these children [34–37]. Moreover, transplantation is often not feasible due to the small size of the child relative to the large donor kidney and is usually recommended after 18 months of age or 10 kg. Growth retardation, which is highly prevalent in these children, further delays transplantation, and thus increases time on dialysis, which in turn increases mortality risk in this already vulnerable population [34, 38]. Nonetheless, relatively good clinical outcomes have been reported, and survival has improved significantly in this group. An international collaboration recently demonstrated a 5-year survival of 76% and a transplant probability of 55%, concluding that relatively good survival may be achieved in neonates despite the high prevalence (73%) of comorbidities [22].

### Sex

No studies have specifically investigated a possible effect of sex on mortality in the pediatric ESRD population, but girls seem to have a higher mortality risk than boys [2]. In the USA, girls >5 years of age on dialysis had a 27% increased mortality risk compared with boys, although this effect was less pronounced in younger children [21]. Girls had an 18% higher cardiovascular-related and a 37% higher infection-related mortality risk compared with boys [39]. A potential explanation was suggested by a European study demonstrating a 23% decreased probability of pre-emptive transplantation in girls compared with boys. This disparity was mostly explained by the fact that girls tended to progress faster to ESRD and by differences in age and primary renal disease distribution. Other potential nonmedical factors, such as patient, parental, and physician attitudes toward transplantation, may also play a role [40].

### Race

Race also affects mortality risk in the pediatric RRT population. In the USA, being black was associated with a 25% higher risk of death compared with being white in first-transplant recipients [24] and a 64% higher risk of death in dialysis patients. The likelihood of transplantation was also lower in both black and Hispanic patients [41]. Furthermore, black children were 1.6 times more likely to die from cardiovascular causes before they reached the age of 30 years compared with white children [42]. The former has been attributed to a higher incidence of hypertension, arrhythmia, cardiomyopathy, and valvular heart disease in blacks [43, 44]. Also, in CKD stages 1–3, black children were more likely to have elevated systolic and diastolic blood pressure than non-black children [45]. In Europe, black and Asian children were less likely to receive a transplant, and Asian children had a 2.5-fold higher mortality risk than white children [46]. Mortality risk was reduced after adjustment for primary renal disease, suggesting that differences in renal disease distribution between races explains part of these disparities.

### Primary renal disease

Congenital anomalies of the kidney and urinary tract (CAKUT) and glomerulonephritis form the most common etiologies of renal disease in children, accounting for at least half of all pediatric ESRD patients [3, 26]. Patients with CAKUT have the best survival probabilities of all primary renal disease groups, although survival varies by etiology [21, 32, 47]. In infants and neonates, those with renal hypo/dysplasia, congenital nephrotic syndrome, polycystic disease, and other/unknown manifestations had a 2- to 4-times increased mortality risk compared with those with obstructive uropathy [23]. Poor patient survival has also been also described in patients with secondary glomerulonephritis, vasculitis, systemic lupus erythematosus, and primary hyperoxaluria [47–49].

### Anthropometry

Children who are either underweight or obese at ESRD onset have an increased mortality risk. In the USA, this U-shaped association was seen in both dialysis and transplant patients, with mortality risk increasing by 26% for every 2 standard deviation (SD) increase or decrease from the 0.5 body mass index standard deviation score (BMI SDS) reference value [50]: in children with a high BMI, increased mortality risk may be due to volume overload, edema, or comorbidity; in underweight children, disease severity and malnutrition may be accountable. Low serum albumin (<3.5 g/dl), a marker for malnutrition or inflammation, was indeed associated with a 90% increased risk of death [51]. Similarly, Ku et al. found that both obese (17% increase) and underweight (26% increase) children were at increased risk of mortality. Interestingly, they found that obese children were less likely to receive a transplant, especially from a living donor, and that this attenuated their increased mortality risk [52].

Growth failure in the pediatric RRT population may reflect disease severity and is associated with increased mortality [53]. In the USA, every SDS decrease in height increased mortality risk by 14%. This effect was particularly evident in children <14 years of age but was similar across treatment modalities [50]. A report from North American Pediatric Renal Trials and Collaborative Studies (NAPRTCS) echoed these results, demonstrating that mortality risk was twice as high in children with a height SDS <2.5 compared with those of normal height [54]. Both short (<3rd percentile) and tall (>3rd percentile) stature at RRT initiation were associated with an increased risk of death, although tall stature was seen only in a small group of white children with elevated BMI (>95th percentile) [55].

### Comorbidity

Extrarenal comorbidity is common in the pediatric ESRD population. The UK Renal Registry reported that at the onset of RRT (2009–2013), 19.3% of pediatric patients had at least one and 9.5% two or more comorbidities. Syndromic diagnosis (8%), developmental delay (7%), and congenital abnormality (7%) were the most frequently reported [56]. Multiple studies show that comorbidity is an important predictor of mortality [21, 57], especially in patients with cognitive (5-year survival probability of 63%), cardiac (73%), and pulmonary (50%) abnormalities [58]. In a single-centre study from the UK, 76% of dialysis patients who died had a comorbid condition, resulting in a 7.5-times increased mortality than those without comorbidities [59]. Several studies show that youngest patients with co-morbid conditions have an increased mortality risk, especially those with pulmonary hypoplasia [60–63].

### RRT modality

**Fig. 1:**
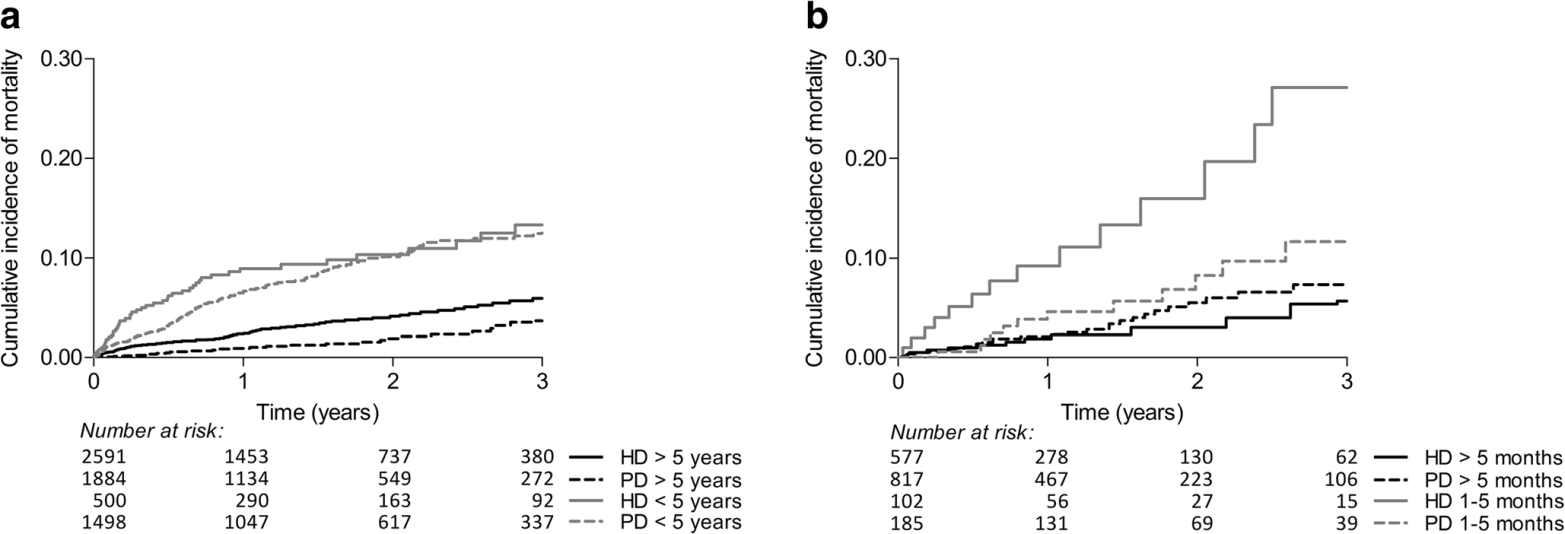
Dialysis modality: **a** age group at renal replacement therapy initiation; –**b** time treated by a nephrologist. Reproduced with minor modification and permission [66].* HD* hemodialysis,* PD* peritoneal dialysis

It is well established that (pre-emptive) renal transplantation offers better survival probabilities than does dialysis [19, 64]. Nonetheless, ∼80% of pediatric patients are either started on dialysis to bridge the preparation time needed for transplantation or will require dialysis after graft loss [3]. Survival comparisons by dialysis modality in a randomized clinical trial (RCT) setting proved extremely difficult [65]. Consequently, survival comparisons remain reliant on observational studies [21, 27, 51, 66–68]. In adults, there seems to be a consistent trend showing survival advantage during the first few years on peritoneal dialysis (PD), especially in younger, healthier, and nondiabetic patients [69–75]. In the pediatric dialysis population, recent registry data from Europe and the USA demonstrate a 21–32% reduced mortality risk in children who are started on PD [21, 66, 67]. In the USA, this treatment effect was only present in children <5 years, whereas in Europe, this effect was less pronounced in children <5 years and absent in infants [21, 66]. Furthermore, European data show that this treatment effect was stronger during the first year of dialysis, in older children, and in children treated by nephrologist for a short period prior to starting dialysis (Fig. 1). As the latter may serve as a proxy for timely referral and speed of disease progression, it may prelude to indication bias due to unmeasured case-mix confounders, as sicker patients are more likely started on hemodialysis (HD) [66].

### Time on RRT

Time spent on dialysis impacts mortality risk, which is highest during the first year of treatment and reflects the intrinsic mortality risk of initiating dialysis. In the USA, mortality rates reach 48 per 1000 patient-years during the first month, peak during the second month at 57, then slowly decrease to 28 during months 9–12. Mortality rates due to cardiovascular disease (CVD) and infection show similar patterns [76].

The length of time living with a functioning graft decreases patient mortality risk. In the USA, in first transplant recipients, mortality was highest during the first post-transplant year, after which the risk decreased (not significantly) by 1% for each additional follow-up year. This effect was stronger for cardiovascular-specific mortality, which decreased by 16% for each follow-up year, suggesting that transplantation has no cumulative negative effect on cardiovascular health in young recipients. However, returning to dialysis after graft failure was associated with a 4.4-fold increase in overall mortality risk and a 7.8-fold increase in cardiovascular mortality risk [39].

### Residual renal function

In adult dialysis patients, a decrease in residual renal function has been associated with an increase in mortality risk [77, 78]. Data is lacking in the pediatric population. Two single-center US studies demonstrated that infants with oligoanuria had a higher mortality risk compared with infants with residual renal function [62, 63], and others have demonstrated a positive effect of residual renal function on growth and nutrition [79–81].

### GFR at RRT initiation

The literature discussing the relationship between glomerular filtration rate (GFR) at dialysis initiation and mortality risk in adults is conflicting [82–84], and this question has not yet been studied in children, although a study from the US found that children with a higher GFR at dialysis initiation had a decreased risk of hospitalization for hypertension and pulmonary edema [85]. A single RCT tackled this question in adults, finding no difference in survival between late and early starters, although the difference (2.2 ml/min/1.73m^2^) in GFR between groups was smaller than anticipated. Nonetheless, dialysis initiation was delayed by 6 months for late starters, which is favorable for both patients and costs [86].

## Causes of death

### CVD and infection-related mortality

CVD and infection-related mortality are the major causes of death in the pediatric RRT population, accountable for ∼30 and 20%, respectively, although rates vary strongly by country, age, race, definition used, and treatment modality [3, 42, 76, 87]. In Europe, infections were the leading cause of death in those on PD and those with a functioning graft, whereas cardiovascular causes dominated in patients on HD [3]. In the USA, a 4.5-times increased risk of CVD death in dialysis compared with transplant patients was reported [42]. In Australia and New Zealand, between 1963 and 2002, CVD accounted for 57% and 43% deaths in HD and PD patients, respectively, and 30% in those with a functioning transplant [19]. Both CVD and infection-related mortality have decreased over recent decades in the USA [21]. Vogelzang et al. studied changes in causes of death in adults on RRT since childhood in The Netherlands, finding that CVD mortality risk decreased by 91% since the 1970s, whereas infection-related mortality risk doubled. The decrease in CVD mortality was attributed to increased awareness among nephrologists of the burden of CVD and a subsequent strict cardiovascular management in these patients [88].

### Malignancy-related mortality

Malignancy-related death occurs more often in transplant recipients than in those on dialysis, likely caused by an impaired tumor immune surveillance due to immunosuppression [2, 39, 89–91]. In Australia and New Zealand, malignancies accounted for 14% of deaths among transplant recipients, compared with only 1% and 2% among patients on HD and PD, respectively, with most deaths occurring after 10 years of RRT [19]. Furthermore, pediatric transplant recipients had a 15- to 30-times increased risk of developing a malignancy compared with the general population [92]. In The Netherlands, 30 years after pediatric transplantation, 41% of survivors had developed cancer and 31% a second de novo cancer during the first year after initial diagnosis. Malignancies were responsible for 13% of all deaths in the cohort, and overall, malignancy was >20-fold higher than in the general population, with a notable increase in risk starting after 20 years of follow-up [93].

## International disparities in survival

As economic welfare is a key determinant of health and access to health services, in low- and middle-income countries, providing chronic RRT is fraught with challenges. The complexity and cost involved in renal care, lack of financial and human resources, different health priorities, and inadequate health infrastructure have obvious consequences for access to RRT and survival probabilities of patients in these countries [94, 95]. In 2010, at least half of the 4.9 million people requiring RRT worldwide died prematurely because they did not have access to treatment [96]. Specifically in children, possibly <10% of those requiring RRT have access to treatment, and most of these preventable deaths occurred in low- and middle-income countries [97]. The few studies available in lower-income countries, where renal registries are often lacking, confirm these disparities. In Jamaica between 2001 and 2006, of all ESRD patients <12 years at diagnosis, 62.5% died due to restricted access to RRT [98]. In a tertiary hospital in South-West Nigeria between 2005 and 2012 the median survival time of 51 admitted pediatric ESRD patients was only 47 days. Of these, 82% had received an acute session of dialysis; however, continuation of RRT was not possible due to financial constraints, likely resulting in death shortly after discharge [99]. In two tertiary hospitals in Vietnam between 2001 and 2005, only 27% of admitted pediatric ESRD patients received RRT. The remainder were treated conservatively due to a lack of financial resources [100]. In a tertiary care hospital in India, 61% of admitted pediatric ESRD patients were either treated conservatively or opted against further treatment due to the high cost of RRT, likely resulting in death [101]. As only a fraction of children requiring RRT globally actually receive treatment, and an equitable and universal provision of costly RRT is unrealistic in the short term, the largest gains in survival are likely to be made by delaying progression of CKD and preventing ESRD [94, 102].

Even among high- and middle-income countries, survival probabilities of pediatric RRT may vary. We recently demonstrated that considerable international variation exists in mortality rates across Europe, mostly attributable to an excess mortality risk for patients treated in several eastern European countries [103]. Most of this variation was explained by disparities in public health expenditure, which determines availability and quality of pediatric renal care services. In addition, differences in a country’s ability to accept and successfully treat the youngest children, who are the most complex and costly to treat, formed an additional source of disparity. Economic constraints were also associated with a lower incidence of RRT [104]. As nonacceptance to RRT implies an underestimation of ESRD mortality rates (as these deaths go unregistered), inequalities in mortality caused by economic constraints will be exacerbated. In addition, considerable country variation persists in transplant rates, donor source, and time on the transplant waiting list, which—given the beneficial effect of transplantation—will affect patient survival indirectly [105].

## Recommendations for long-term follow-up through adulthood

The increased mortality risk of pediatric-onset ESRD remains in adulthood, with life expectancy reduced by 40–50 years in dialysis patients and 20–30 years in transplant patients [14]. CVD is highly prevalent amongs young adults after lengthy exposure to RRT but is reversible [106–109]. Strict monitoring of CVD and intensified antihypertensive and antilipemic therapy should therefore be a priority. As most pediatric-onset ESRD patients will have received a transplant prior to transitioning to adult care, continued compliance to immunosuppression regimens is of the upmost importance, especially given that up to 53% of adolescents are noncompliant [110–112]. Moreover, due to prolonged exposure to immunosuppression in these patients, adult nephrologists should be attentive to the increased risk of infections and the development of skin cancers 10–15 years posttransplantation.

## Knowledge gaps

National and international registries for pediatric RRT have been instrumental in describing survival and establishing factors associated with mortality. However, data from middle- and lower-income countries remain scarce. The forthcoming International Pediatric Nephrology Association (IPNA) registry aims to consolidate existing registry data and fill in the gaps by collecting global data [113]. Worldwide reporting of pediatric RRT is essential to determine international disparities in treatment modalities and mortality rates, increase awareness of these disparities, and provide evidence to advocate policy change and inform budgetary decisions at various levels of government.

Furthermore, although associations between mortality and various patient- and treatment-related factors have been studied in the adult RRT population, simple extrapolation of these results to children is often not valid given the differences in disease etiology and progression. Small samples sizes and a low number of adverse events often impede epidemiological research. Nonetheless, with continued support and commitment, the volume of registry data will increase over time, hopefully enabling studies to fill the knowledge gaps concerning determinants of mortality, specifically in the pediatric RRT population [114].

## Limitations

Several factors limited our ability to investigate mortality risk in the pediatric RRT population. First, children with ESRD who are not accepted for RRT or who died prior to treatment initiation are not registered. Second, patients are frequently lost to follow-up in registries when transferred to adult care, precluding registration of premature death during (early) adulthood. Third, studies often focus on mortality risk of either dialysis or transplantation instead of throughout the entire RRT trajectory. Lastly, in contrast to adult patients, virtually all children with ESRD are considered transplantable, and thus long-term dialysis studies are scarce and subject to negative selection of nontransplantable patients.

## Summary

- Patient survival has improved substantially over recent decades in both dialysis and transplant populations, and although the youngest patients bear the highest mortality risk, they also show the greatest improvement in survival over time.
- Patient survival is multifactorial, largely dependent on access to treatment, country health expenditure, disease etiology, age, transplant feasibility, growth failure, sex, BMI, race, and presence of comorbidities.
- Although comparisons between dialysis modalities are hindered by selection bias and residual confounding, patients initiated on PD seem to have an initial survival advantage over those initiated on HD.
- Global disparities persist in the provision of RRT and outcomes in the pediatric ESRD population, even among middle- and higher-income countries.

## Questions (answers are provided following the reference list)

1. What is roughly the 5-year survival rate for an average pediatric patient starting RRT in a developed country?

- 90%
- 80%
- 70%
- 60%

2. Which patient group has seen the greatest improvement in survival over the past decade?

- Youngest patients
- Pubertal patients
- Patients with a CAKUT diagnosis
- Patients receiving a pre-emptive transplant

3. Which patient population has an increased risk of cardiovascular mortality?

- Black patients
- Youngest patients
- Hemodialysis patients
- All of the above

4. What is roughly the 20-year survival rate for an average pediatric patient initiating RRT with a pre-emptive transplant in Europe?
  - 75%
  - 80%
  - 90%
  - 95%
5. Which factor(s) are responsible for the global disparities in treatment outcomes for children?

- Capacity to provide RRT to the youngest patients
- Macroeconomic constraints
- Financial burden for patient/family
- All of the above
